# ICTV Virus Taxonomy Profile: *Nimaviridae*

**DOI:** 10.1099/jgv.0.001248

**Published:** 2019-03-29

**Authors:** Han-Ching Wang, Ikuo Hirono, Mary Beth Bacano Maningas, Kunlaya Somboonwiwat, Grant Stentiford, ICTV Report Consortium

**Affiliations:** ^1^​ Department of Biotechnology and Bioindustry Sciences, National Cheng Kung University, Tainan, 701, Taiwan, ROC; ^2^​ International Center for the Scientific Development of Shrimp Aquaculture, National Cheng Kung University, Tainan, 701, Taiwan, ROC; ^3^​ Graduate School of Marine Science and Technology, Tokyo University of Marine Science andTechnology, Tokyo, 108-8477, Japan; ^4^​ Department of Biological Sciences, University of Santo Tomas, España, Manila, 1015, Philippines; ^5^​ Department of Biochemistry, Faculty of Science, Chulalongkorn University, Bangkok, 10330, Thailand; ^6^​ Pathology and Molecular Systematics Team, Centre for Environment, Fisheries andAquaculture Science (Cefas), Weymouth, DT4 8UB, UK

**Keywords:** *Nimaviridae*, ICTV Report, taxonomy, white spot syndrome virus

## Abstract

The family *Nimaviridae* includes the single species *White spot syndrome virus*, isolates of which infect a wide range of aquatic crustaceans and cause substantial economic losses. Virions are ellipsoid to bacilliform with a terminal thread-like extension. The circular dsDNA genome is 280–307 kbp with several homologous repeat regions. More than 80 structural and functional proteins have been characterized from 531 ORFs. White spot syndrome is a highly lethal, contagious disease associated with white spot syndrome virus infection of shrimps. This is a summary of the International Committee on Taxonomy of Viruses (ICTV) Report on the family *Nimaviridae*, which is available at www.ictv.global/report/nimaviridae.

## Abbreviation

Hr, homologous repeat.

## Virion

**Fig. 1.**
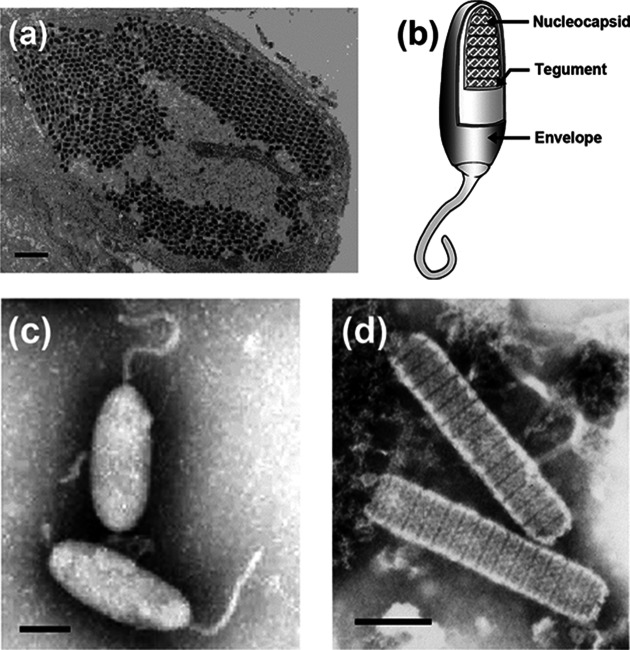
(a) Shrimp stomach epithelium infected with white spot syndrome virus showing the parallel arrangement of virions in the nucleoplasm (bar, 1 µm) (courtesy of Don Lightner). (b) Schematic illustration of the structure of a typical *whispovirus* virion. (c) Negative-contrast electron micrograph of intact white spot syndrome virions; bar, 100 nm (courtesy of Marielle van Hulten). (d) Negative-contrast electron micrograph of white spot syndrome virus nucleocapsids; bar, 100 nm (courtesy of Don Lightner).

Virions are enveloped, ellipsoid-to-bacilliform particles of 70–170 nm in width and 210–420 nm in length (Table 1, (Fig. 1). A thread-like projection (or extension) is present at one end of many virions which, from the Greek word for thread (*Níma*), gives the family its name. The virion consists of an inner, rod-shaped, striated nucleocapsid with a tight-fitting capsid layer, an intermediate tegument layer and an outer lipid-containing trilaminar envelope [1]. Virion particle lipids are apparently derived from host-cell nuclei. The purified nucleocapsid measures 50–80 nm in width and 300–420 nm in length. Proteomic analysis indicates that the virions contain more than 40 structural proteins [1, 2].

**Table 1.** Characteristics of members of the family *Nimaviridae*

Typical member: white spot syndrome virus-CN (AF332093), species *White spot syndrome virus*, genus *Whispovirus*	
Virion	Ellipsoid-to-bacilliform virion consisting of envelope, tegument, rod-shaped nucleocapsid and, sometimes, a thread-like terminal extension; 70–170 nm×210–420 nm, containing >40 structural proteins
Genome	A single circular dsDNA of 280–307 kbp with nine internal homologous repeat regions
Replication	Formation of the nucleocapsid and assembly of the intact virion occur in the nucleus; only virus protein translation occurs in the cytoplasm
Translation	Most virus mRNAs are capped, polyadenylated and translated via the canonical cap-dependent pathway; some genes, including structural genes, are expressed via mRNAs translated by the canonical cap-independent pathway using internal ribosome entry site elements
Host range	Wide range of crustaceans (order Decapoda) from marine, brackish or freshwater sources
Taxonomy	Single genus and species

## Genome

**Fig. 2.**
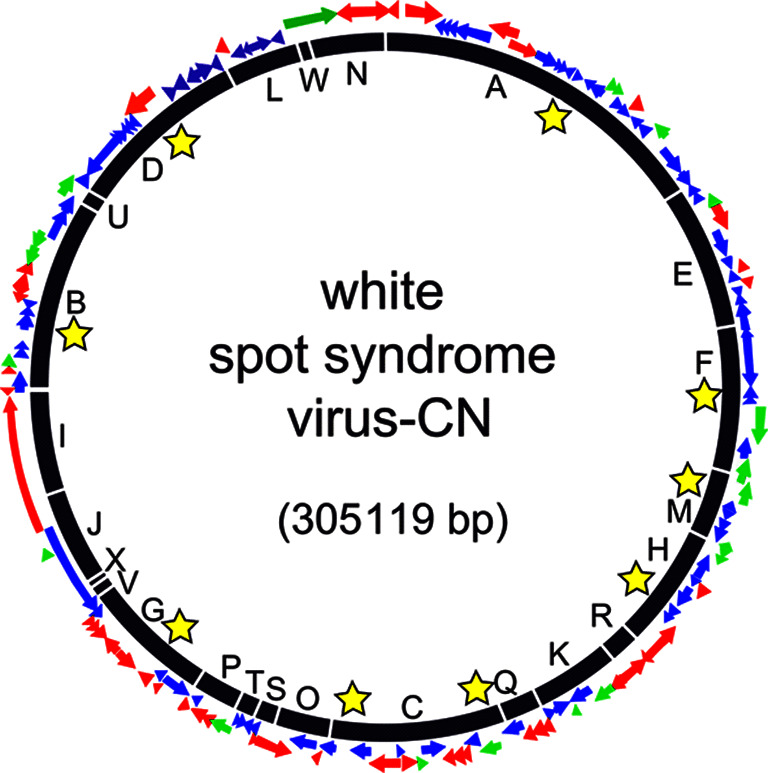
Schematic map of the circular dsDNA genome of white spot syndrome virus-CN (AF332093). ORFs are coloured red (structural proteins), green (functional proteins) and blue (unknown function). Yellow stars indicate homologous repeats. BamHI restriction fragments are labelled A–X.

The genomes of isolates from China (AF332093), Thailand (AF369029), Taiwan (AF440570), Korea (JX515788), Mexico (KU216744), India (MG702567) and Australia (MF768985) are single circular dsDNA molecules of 280–307 kbp with a mean G+C content of approximately 41 %. The genome of white spot syndrome virus-CN (AF332093) contains 531 ORFs of 60 codons or more, although only 181 ORFs are likely to encode functional proteins [3]. The white spot syndrome virus genome contains nine homologous repeat (Hr) regions (Fig. 2). The number of imperfect palindromic repeats (250 bp) within an Hr varies among isolates [4].

## Replication

Virus is transmitted by cannibalism of diseased individuals or via water passing through the gills. It can also be transmitted vertically from adults to offspring, either by being released from non-viable infected eggs or from the supporting cells in ovarian tissue. The transcription of virus mRNA, the replication of virus genomic DNA, the formation of nucleocapsids and the assembly of intact virion particles all take place in the nucleus; virus proteins are translated in the cytoplasm. Transcription of virus genes is temporally regulated, and four main classes of genes are assigned according to the time of mRNA expression: immediate early, early, late and very late genes. Most mRNAs are capped, polyadenylated and translated via the canonical cap-dependent pathway. Some genes, including structural genes, are expressed via mRNAs translated by the canonical cap-independent pathway using internal ribosome entry site elements. Splicing of mRNAs has not been observed [5].

## Taxonomy

Phylogenetic analysis of DNA polymerase sequences shows that white spot syndrome virus is highly distinct from other dsDNA viruses. Isolates identified in crustaceans in China, Japan, Korea, Southeast Asia, South Asia, the Middle East, Australia and the Americas show little genetic diversity (e.g. DNA polymerase amino acid sequences are approximately 99 % identical). Thus, all isolates are classified in the single species *White spot syndrome virus* in the genus *Whispovirus*. The extremely wide host range and very low level of genetic polymorphism suggest that white spot disease is a relatively recent epizootic.

## Resources

Full ICTV Report on the family *Nimaviridae*: www.ictv.global/report/nimaviridae.
